# Co-Inoculation of Endophytes *Bacillus siamensis* TUR07-02b and *Priestia megaterium* SMBH14-02 Promotes Growth in Rice with Low Doses of Nitrogen Fertilizer

**DOI:** 10.3390/plants12030524

**Published:** 2023-01-23

**Authors:** Winston Franz Rios-Ruiz, Ciceron Tuanama-Reátegui, Gamaniel Huamán-Córdova, Renzo Alfredo Valdez-Nuñez

**Affiliations:** 1 Laboratorio de Microbiología Agrícola, Departamento Académico Agrosilvopastoril, Facultad de Ciencias Agrarias, Universidad Nacional de San Martín, Tarapoto 22202, Perú; 2 Departamento Académico de Ciencias Básicas, Facultad de Ingeniería, Universidad Nacional de Barranca, Barranca 15169, Perú

**Keywords:** efficient use of nitrogen, phosphate solubilization, bioinoculants, auxins, rice

## Abstract

Multiple biotic and abiotic factors influence rice cultivation. These factors limit productivity and yield, as well as an irrational use of agrochemicals in rice cultivation. A sustainable alternative is using selected growth-promoting microorganisms to increase nutritional efficiency. In the present study, the direct mechanisms of growth promotion in two strains of *Bacillus*, three strains of *Priestia,* and two strains of *Burkholderia* endophytes of rice were characterized. *Bacillus siamensis* TUR07-02b and *Priestia megaterium* SMBH14-02 were selected to promote Oryza sativa var’s growth. “Bellavista” was used at different doses (50, 75, and 100%) of mineral nitrogen (N) using a randomized block design by quintuplicate. Both strains, SMBH14-02 and TUR07-02b, presented outstanding promoter characteristics, including auxin production (123.17 and 335.65 μg mL^−1^, respectively) and biological nitrogen fixation capacity. Similarly, *B. siamensis* TUR07-02b could solubilize phosphate-Ca (20.94 μg mL^−1^), cellulases, and pectinases. Under greenhouse conditions, co-inoculated plants receiving 75% of the total dose of mineral nitrogen showed increased agronomic parameters in relation to panicle length, grains per panicle, grain yield, and harvest index by 25.0, 30.7, 39.5, and 12.5%, respectively, compared to the 75% fertilized treatment without inoculation. The strains of *B. siamensis* TUR07-02b and *P. megaterium* SMBH14-02 are potential microbial resources in the formulation of new inoculants to reduce the use of nitrogenous fertilizers. Thus, agronomic validation of the inoculant consortium at the field level will be an essential step in providing an alternative for the sustainable management of rice cultivation and increased productivity of rice farmers in the San Martín region.

## 1. Introduction

Rice is one of the most consumed cereals on the planet. Approximately 1.62 × 10^8^ hectares are cultivated worldwide, producing approximately 7.55 × 10^8^ tons of rice grain. Although the production of Latin America represents only 4.68% of this quantity, the crop is of paramount importance for food security and daily carbohydrate intake in the Latin American population [1].

The agronomic potential of rice is limited by abiotic factors, such as high temperatures, water stress, and salinity [2,3], and biotic factors such as pests and diseases [4], but also by nitrogen levels in the soil. Nitrogen plays a fundamental role in producing chlorophyll and other cellular components, such as amino acids and nucleic acids, which are involved in the productivity and yield of rice [5]. In addition, a series of nitrate reductases finely regulate nitrogen assimilation in response to environmental conditions [6].

Rice assimilates nitrogen in the forms of ammonium (NH_4_^+^) or nitrate (NO_3_^−^), with one of the primary sources being urea [7]. However, when nitrogen is limited in the soil due to microbiological factors, environmental changes, and cultural practices, farmers are forced to use nitrogen fertilizers. In the worst-case scenario, excessive use of nitrogenous fertilizers releases greenhouse gases, contaminates water bodies, and damages sil biology [8,9].

The use of microorganisms with the capacity to promote plant growth constitutes a sustainable option for increasing crop production. For rice cultivation, various microorganisms that use direct growth-promoting mechanisms, such as biological nitrogen fixation, indole acetic acid production, inorganic phosphate solubilization, and the presence of 1-aminocyclopropane-1-carboxylate deaminase (ACCD) activity, among others, have been reported [10], including through the use of multi-omics technologies [9,11].

Works related to applying *Bacillus* inoculants to reduce the use of fertilizers and increase rice productivity have also been reported [12,13,14,15].

*B. siamensis* is a recognized plant growth-promoting bacterium, which promotes the health and growth of plants, reduces oxidative stress, increases antioxidant enzymatic activity in wheat [16], and improves nitrogen use efficiency in *Capsicum annuum* [17]. The production of phytohormones is the leading direct PGPR mechanism [18], and volatile components with antifungal activity are the indirect PGPR mechanism [19,20]. It was reported that the rice endophyte *B. siamensis* can promote root development by producing volatile compounds independent of the hormonal pathway mediated by auxins, ethylene, or jasmonic acid. The growth promotion capacity of *P. megaterium* in various crops, such as rice, is better known [21]. Among the main growth promotion mechanisms described, the solubilization of phosphates by organic acids is reported [22], the production of auxins and cytokinins [23], as well as the control of phytopathogens, from the production of antifungals, volatile compounds, and inactivation of acyl-homoserine lactones (quorum quenching) [24].

The strains under study were isolated as endophytes from different organs of rice plants grown in Northern Peru and previously published [25]. *P. megaterium* SMBH14-02 and *B. siamensis* TUR07-02b were able to inhibit the growth of *B. glumae* THT, produce siderophores, and show slight resistance to toxoflavin (30 µg mL^−1^). However, the inoculation of endophytes, which shows synergy between them and present rice growth-promoting characteristics under low doses of nitrogenous fertilizers, has not yet been reported.

We hypothesize that co-inoculation of endophytic *Bacillus* and *Priestia* bacteria can promote the growth of rice plants with low doses of nitrogen fertilizer. Therefore, the objective of the present work was to evaluate the effects of inoculation with strains of *Bacillus*, *Priestia,* and *Burkholderia* in promoting the growth of rice of the “Bellavista” variety under different doses of nitrogen fertilization and in greenhouse conditions.

## 2. Results

### 2.1. Strains under Study and Verification of Endophytic Colonization

**Figure 1:**
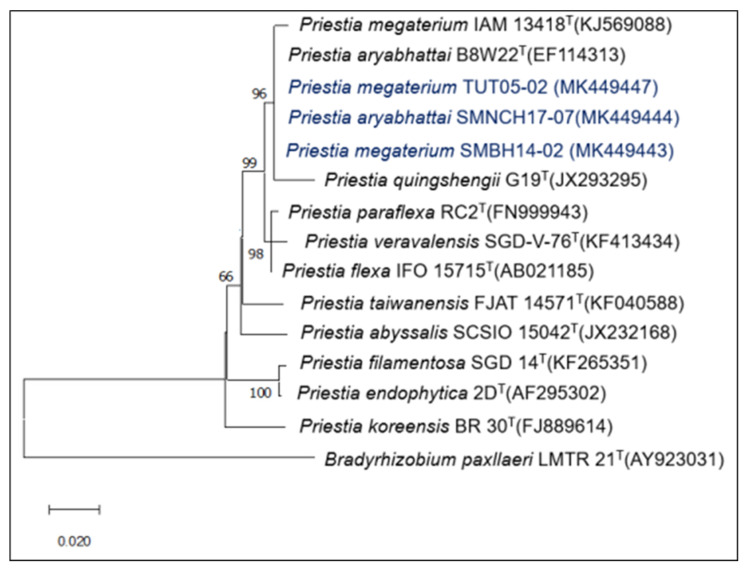
Neighbor-joining phylogenetic tree based on 16S rDNA gene sequences (1300 positions) showing the relationships among endophytes *Priestia* species isolated from rice in Northern Peru and closely related species of the genus *Priestia*. A bootstrap value indicates the significance of each branch (as percentage) calculated for 1000 subsets (only values greater than 50% are indicated). Bar, two substitutions per 100 nucleotide positions. The 16S rDNA sequence of *Bradyrhizobium paxllaeri* LMTR-21^T^ was used as an outgroup.

**Figure 2:**
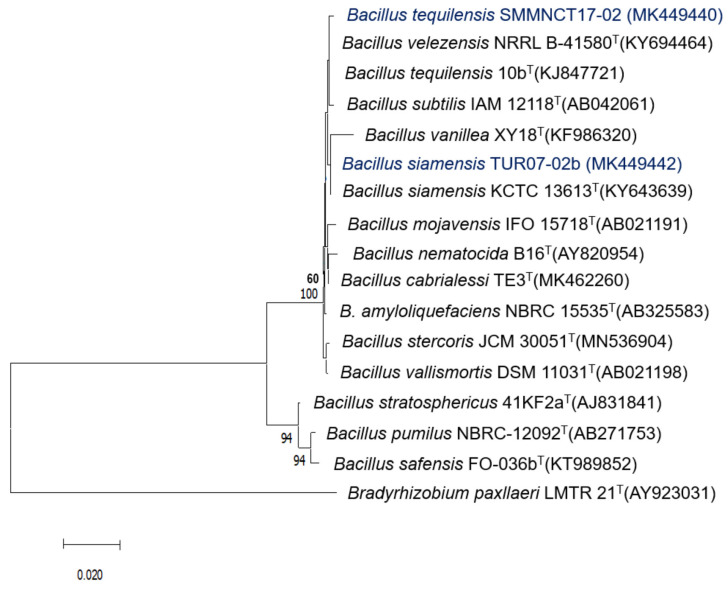
Neighbor-joining phylogenetic tree based on 16S rDNA gene sequences (1292 positions) showing the relationships among endophytes *Bacillus* species isolated from rice in Northern Peru and closely related species of the genus *Bacillus*. A bootstrap value indicates the significance of each branch (as a percentage) calculated for 1000 subsets (only values greater than 50% are indicated). Bar, two substitutions per 100 nucleotide positions. The 16S rDNA sequence of *Bradyrhizobium paxllaeri* LMTR-21^T^ was used as an outgroup.

**Figure 3:**
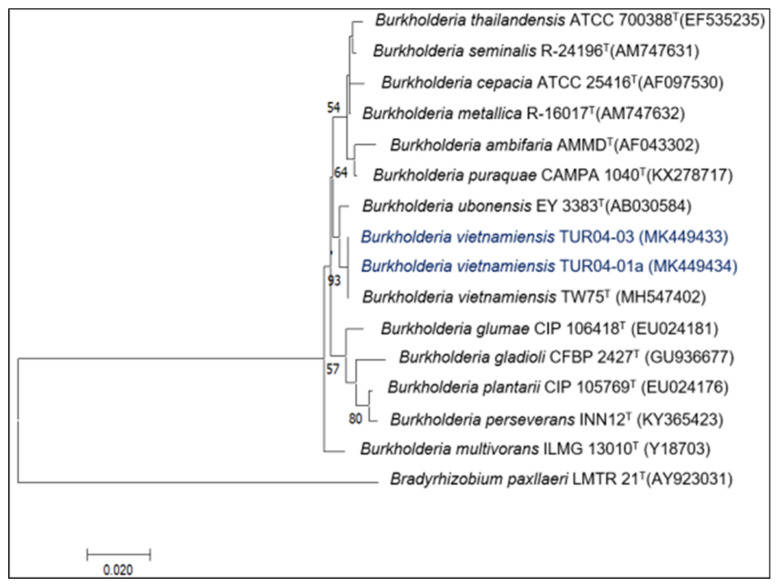
Neighbor-joining phylogenetic tree based on 16S rDNA gene sequences (1258 positions) showing the relationships among endophytes *Burkholderia* species isolated from rice in Northern Peru and closely related species of the genus *Burkholderia*. A bootstrap value indicates the significance of each branch (as percentage) calculated for 1000 subsets (only values greater than 50% are indicated). Bar, two substitutions per 100 nucleotide positions. The 16S rDNA sequence of *Bradyrhizobium paxllaeri* LMTR-21^T^ was used as an outgroup.

The seven bacterial strains used in the present study were isolated as rice endophytes and identified as *Priestia* (MK449443, MK449444, MK449447) (Figure 1), *Bacillus* (MK449440, MK449442) (Figure 2), and *Burkholderia* (MK449433, MK449434) (Figure 3) [25]. The endophytic behavior of the strains was verified by counting the bacterial populations colonizing the root and stem (CFU g^−1^ fresh weight) compared to the endophytic bacterial populations of the non-inoculated control treatment (Table 1). All the inoculated root and stem endophytic bacterial counts were higher than those in the control treatment. The *Bacillus tequilensis* strain SMNCT17-02 presented significantly higher counts of endophytic bacterial populations in the root and stem than the other strains under study, including the control endophytic bacteria *B. vietnamiensis* la1a4. At 7 days post-inoculation, the populations of *B. tequilensis* SMNCT17-02 were lower at the root level (5.71 CFU g^−1^ fresh weight) in relation to the stem (6.11 CFU g^−1^ fresh weight). The endophytic colonization of the *Bacillus* and *Priestia* genera in the root (2.60 to 5.71 CFU g^−1^ fresh weight) and stem (3.04 to 6.11 CFU g^−1^ fresh weight) was, on average, higher than that observed in root (3.58 to 3.86 CFU g^−1^ fresh weight) and stem (3.93 to 3.99 CFU g^−1^ fresh weight) for the *Burkholderia* genus.

### 2.2. Evaluation of Plant-Growth-Promoting (PGP) Bacterial Characteristics

All the strains under study produced indolic compounds directly proportional to tryptophan supplementation in the culture medium (Table 2). Under basal conditions (0 ppm tryptophan), the production of indolic compounds by *B. siamensis* strain TUR07-02b was significantly higher than that by the other strains. All strains used in this study produced indole acetic acid (IAA) when the culture medium was supplemented with 300 µg of tryptophan mL^−1^. The *B. siamensis* strain TUR07-02b could produce high concentrations of IAA when supplemented with 600 µg of tryptophan mL^−1^.

The *B. siamensis* strain TUR07-02b stood out for its moderate ability to solubilize tricalcium phosphate but not iron phosphate or aluminum phosphate (Table 3). On the other hand, the *B. tequilensis* SMNCT17-02 strain stood out for its ability to solubilize aluminum phosphate. No strains with solubilizing activity associated with iron phosphate were found.

**Figure 4:**
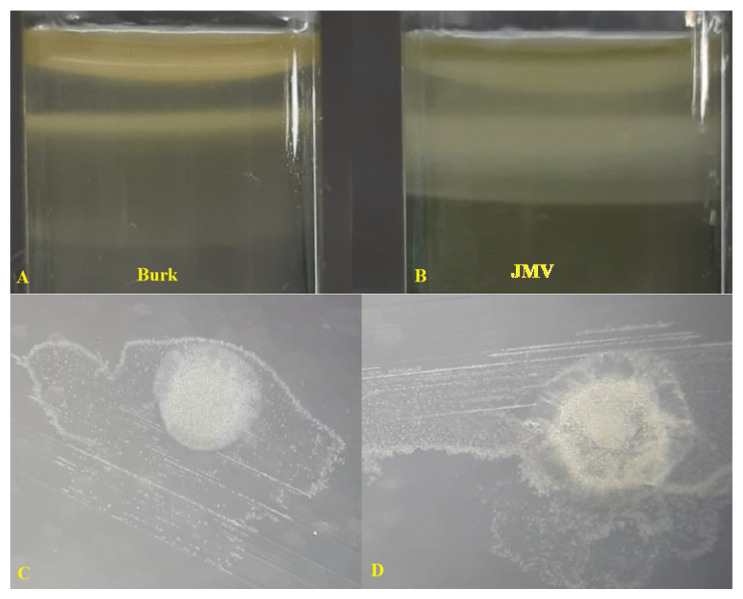
Phenotypic characteristics in semi-solid medium (**A**,**B**) and morphocolonial characteristics in solid medium (**C**,**D**) of the *Bacillus tequilensis* SMNCT 17-02 strain grown in two nitrogen-free culture medias.

**Figure 5:**
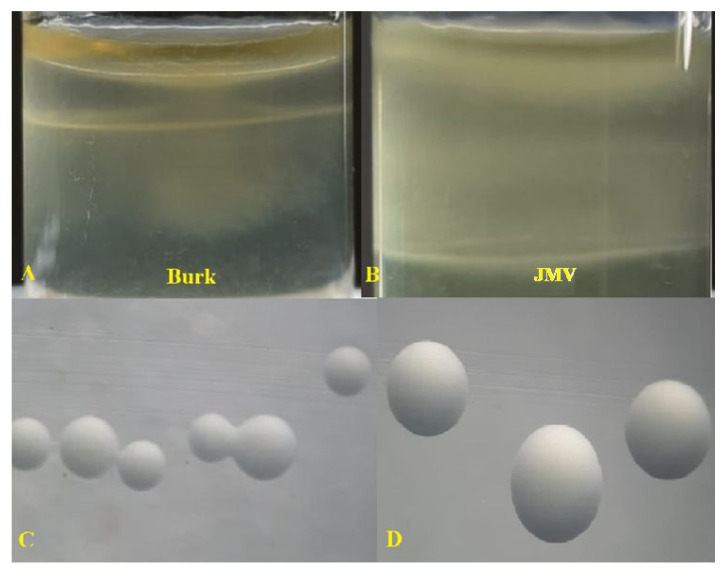
Phenotypic characteristics in semi-solid medium (**A**,**B**) and morphocolonial characteristics in solid medium (**C**,**D**) of the *Burkholderia vietnamiensis* TUR 04-03 strain grown in two nitrogen-free culture medias.

Regarding nitrogen fixation, all the strains under study were able to grow in a semi-solid medium containing mannitol and glucose as carbon sources (Table 3 and Figure 4 and Figure 5).

Regarding the production of cellular enzymes, *B. siamensis* TUR07-02b and *P. megaterium* SMBH14-02 presented cellulase and pectinase activity, while *P. megaterium* SMBH14-02 presented only pectinase activity (Table 3), although none of the strains were able to produce chitinases.

### 2.3. Growth-Promotion Experiments

**Figure 6:**
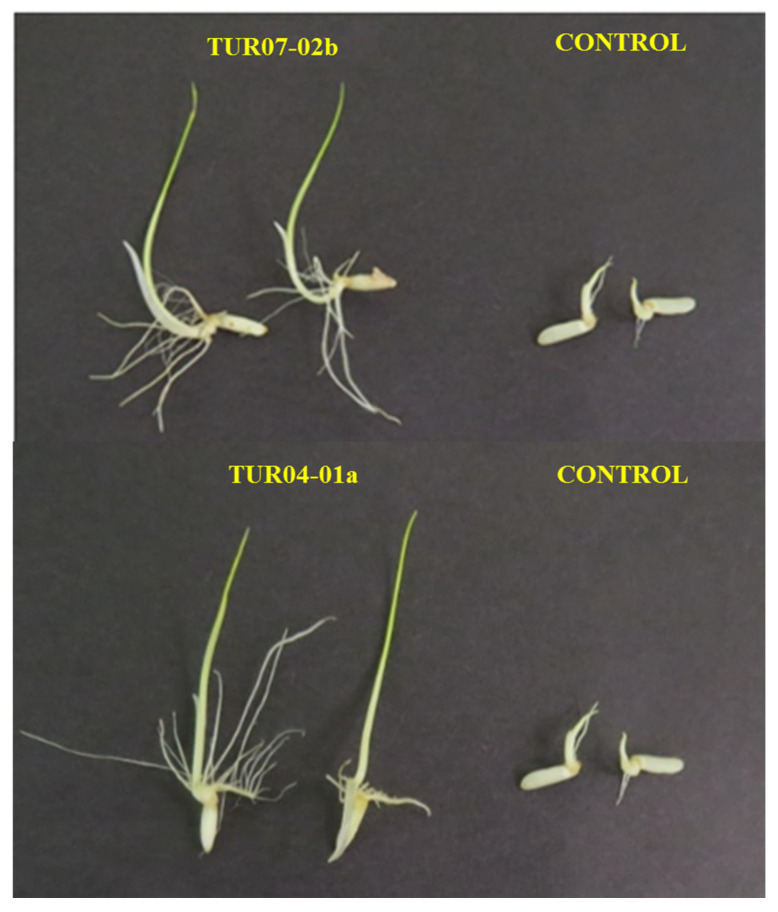
Effect of inoculation of *B. siamensis* TUR07-02b and *B. vietnamiensis* TUR04-01a on *Oryza sativa* var. “Bellavista”, compared to the control without inoculation.

In the first stage, the effect of inoculation with the endophytic strains of *Bacillus* and *Burkholderia* on the percentage of rice germination was evaluated. The effect of inoculation with *P. megaterium* SMBH14-02 and *B. siamensis* TUR07-02b strains on the germination percentage of the “Bellavista” rice variety was superior and statistically significant, with increases in germination percentage between 12.2 and 13.4% compared to the control without inoculation (Table 4 and Figure 6).

In the second experiment, the rice plants were inoculated individually and in a consortium with the two strains that significantly promoted the percentage of germination in rice (*B. siamensis* TUR07-02b and *P. megaterium* SMBH14-02). These strains were compatible with each other, showing no growth inhibition at an in vitro level. About the length of the panicle, the co-inoculated treatments receiving doses of 50% (30.38 mm), 75% (30.90 mm), and 100% (32.27 mm) of nitrogen fertilization were statistically significantly increased (*p* < 0.05), compared to treatments with simple inoculation and chemical treatments without inoculation (Table 5). The panicle length in the co-inoculated treatment receiving 75% of the fertilizer nitrogen dose (30.90 mm) was 25.0% higher than that achieved in the chemical control treatment (75%) (24.72 mm). The number of grains per panicle in the co-inoculated treatment receiving 75% of the nitrogen dose (150.80) was statistically significantly increased (*p* < 0.05) compared to the treatments with simple inoculation and chemical treatments without inoculation (Table 5). This treatment was superior by 30.7% compared to the treatment fertilized with 75% nitrogen. On the other hand, grain yield was statistically significantly increased in co-inoculated treatments receiving 50% (4.06 g plant^−1^) and 75% (4.34 g plant^−1^) of nitrogen fertilization (*p* < 0.05), compared to the treatments with simple inoculation and chemical treatments without inoculation (Table 5). The grain yield was higher by 31.4 and 39.5% compared to the treatment fertilized with 50 and 75%, respectively. Another critical parameter derived from straw and grain yield is the harvest index (Table 5). This parameter was higher in the co-inoculated treatments receiving 50, 75, and 100% of the nitrogen dose compared to the treatments with simple inoculation and chemical treatments without inoculation. The harvest index was 12.5% higher in the co-inoculated treatment receiving 75% of the nitrogen dose compared to the fertilized treatment. The co-inoculated plants receiving nitrogen fertilization (50–100%) presented harvest indices between 21.20% and 22.16%, which were higher than the rest of the individually inoculated treatments (12.89–18.23%) and the chemical control (13.34–20.01%). The co-inoculation of *B. siamensis* TUR07-02b and *P. megaterium* SMBH14-02 in the “Bellavista” variety promoted an increase in grain production.

## 3. Discussion

### 3.1. Strains under Study and Verification of Endophytic Colonization

*Bacillus* and *Priestia* endophytes colonize different organs in rice, such as the root, stem, leaves, and grain, showing a great diversity of species and participating as promoters of plant growth [14] and in the degradation of agrochemicals [26], suppression of diseases [15,27], and induced systemic resistance [28]. The genus *Burkholderia* has been reported as a frequent endophyte in rice at the root level [29,30]. *Burkholderia vietnamiensis* is considered a model growth-promoting bacterium in rice [31], through improving the efficient use of mineral nitrogen [10,13] and biocontrol [32].

The endophytic style of a strain can be verified by direct observation using phase-contrast microscopy, immunofluorescence, or direct counting of the tissue under study. It has been reported that *Bacillus*, *Priestia,* and *Burkholderia* are common endophytes in rice and that the frequency of both naturally fluctuates between 10^2^ and 10^6^ CFU g^−1^ of fresh weight in seeds [33,34], coinciding with what was reported in our study. This colonization is intercellular but not intracellular in epidermal cells and substomatal cavities in the leaves and stem [35].

The population counts of *B. tequilensis* SMNCT17-02 found at the root and stem level in this study were different from previously reported values of endophytic colonization of *Bacillus oryzicola* (*B. velezensis*) YC7007, being higher in the root (6.53 CFU g^- 1^) in relation to the stem (5.70 CFU g^−1^) [36]. This difference of concentration used of an inoculum 2 × 10^7^ CFU mL^−1^ compared to the present study of 10^9^ CFU mL^−1^ could explain this difference and the number of days to evaluation (8 days after the inoculation) and intrinsic characteristics of the species or strain. Furthermore, another study reported that the colonization of *Burkholderia* sp. AG1004 reached populations of 10^4^ CFU g^−1^ [37], similar to our study. In contrast, populations higher than 10^6^ CFU mg^−1^ have been reported in the roots of rice seedlings of varieties IR64 (Indica) and Nipponbare (Japonica) at 7 days post-inoculation with *B. vietnamiensis* LMG10929, suggesting differences at the level of rice genotype and bacterial strain [31].

It is essential to highlight that the endophytic colonization of our isolates was intermediate. Although, strains such as *B. siamensis* TUR07-02b and *P. megaterium* SMBH14-02 presented an optimal growth promotion capacity in rice and an intermediate level of endophytic colonization. Bioprospecting of strains with greater endophytic colonization capacity is necessary considering that the ability of PGPR to promote plant growth depends on their root colonization capacity. Colonization research is important for understanding plant-microorganism interactions [38], especially in their performance as future inoculants at the field level.

### 3.2. Evaluation of PGP Bacterial Characteristics

The production of auxins is a common feature among endophytic bacteria of rice, especially in the genera *Bacillus* [14,39] and *Burkholderia* [30], which is why it is considered a feature in the selection of competent endophytic bacteria as promotion agents in rice [40]. In the present study, both *B. siamensis* TUR07-02b and *P. megaterium* SMBH14-02 produced indolic compounds in amounts proportional to the application of tryptophan in the culture medium. Likewise, these strains produced a higher concentration of IAA in the media supplemented with increasing concentrations of tryptophan, which explains their superiority over other strains under study

It has been reported the endophytic rice strain *B. aryabhattai* MN1 is a good producer of indole compounds in a medium supplemented with 1000 µg of tryptophan mL^−1^ [41]. The differences in IAA production could be because some PGP bacteria can degrade IAA [42,43]. IAA, stimulating the overproduction of root hairs and lateral roots, increases the production of root exudates, generating a better rhizospheric effect [40]. This rhizospheric effect causes changes in the soil’s organic matter that are necessary for the adequate development of plants by playing an essential role in regulating the carbon and nitrogen cycle [44].

Phosphorus is a limiting element in agriculture; despite being present in sufficient quantities in the soil, it is not available for plants [45]. The solubilization of phosphates by endophytic microorganisms has been described as more effective than that of non- endophytic and facultative microorganisms [46,47]. It was previously reported that the tricalcium-phosphate-solubilizing activity of the endophytic rice strain HS-S05 of *B. aryabhattai* was only 0.6 µg mL^−1^ [48]; similarly, minimum values of tricalcium phosphate solubilization were reported when evaluating the AS6 strain of *B. aryabhattai* isolated from the rice rhizosphere [49]. The present study found that *B. siamensis* TUR07-02b solubilized tricalcium phosphate performs well in slightly alkaline soils. In contrast, *B. tequilensis* SMNCT17-02, which solubilized aluminum phosphate, could perform better in acid soils. Although the solubilization of phosphates complexed with aluminum has been described in different bacterial genera [50], there are few reports of solubilizing activity associated with aluminum phosphate in the genus *Bacillus* [51]. In our study, the only aluminum-phosphate-solubilizing activity was found in *B. tequilensis* SMNCT17-02. Endophytic tricalcium-phosphate-solubilizing activity can be documented via the production of organic acids, among which gluconic acid is distinguished [46,47].

The ability to fix atmospheric nitrogen has been widely reported in the genera *Bacillus* [52,53] and *Burkholderia* [54,55], as was observed in the strains studied in this work. Moreover, the ability of *B. vietnamiensis* to fix atmospheric nitrogen contributes minimally to its mutualistic performance with rice [13].

The production of extracellular enzymes in endophytic bacteria is related to the penetration and dispersal of endophytic bacteria inside the plant [56]. In the present study, both the SMBR14-02 and TUR07-02b strains produced cellulase and pectinase enzymes. The activity of these enzymes can explain the endophytic behavior of the strains since the invasion process requires the degradation of the cell walls [56]. Although none of the strains could produce chitinases, a potential “in vitro” antagonistic action against phytopathogenic fungi is not excluded.

### 3.3. Growth-Promotion Experiments

Among the phytobeneficial endophytic species in rice cultivation [52], *B. velezensis* [57], *B. megaterium* [15], *B. siamensis* [20], and *B. tequilensis* [58] are highlighted. In the case of *Burkholderia*, its use is restricted in agriculture despite its potential for growth promotion and biocontrol because some strains that are members of the *B. cepacea* complex are opportunistic human pathogens [59]. Regarding the effect of inoculation with endophytic bacteria on the germination percentage, some authors have reported higher germination percentage values (48.4%) compared to control treatments [60]. Similarly, it was reported that *B. tequilensis* JN-369 promoted the germination process of rice seeds, but this was not significant [61]. Among the factors that affect the germination rate of rice at the time of inoculation are the initial population of the inoculum and the bacterial physiological state [62].

The parameters related to panicle length [63,64], number of panicles per plant, and grains per panicle [63] are suitable agronomic indicators of grain yield when evaluating the effectiveness of bacterial consortia at the greenhouse and field level [65,66]. In our study, panicle lengths in co-inoculated treatments receiving nitrogen fertilization were significantly greater than those with simple inoculation and chemical treatments without inoculation. It was reported previously that co-inoculation whit the phosphate-solubilizing rhizobacteria *Pseudomonas aeruginosa* and *Bacillus subtilis* increased panicle length by 63.9% compared to a control treatment [65]. There is evidence that panicle length is a good indicator of panicle architecture and grain yield, which is associated with the LP1 locus on rice chromosome 9 [64,67].

Concerning the number of grains per panicle, in our study, better results were obtained in the treatment co-inoculated with the strains *B. siamensis* TUR07-02b and *P. megaterium* SMBH14-02 compared to the treatments with simple inoculation and the control with chemical fertilization. A similar result was reported when inoculating two rice varieties with *Enterobacter hormaechei* AM122 and *Lysinibacillus xylanilyticus* DB25, both in simple inoculation and in consortium, and it was reported that the consortium significantly increased the number of grains per panicle compared to the simple inoculation treatment and the control [66].

There is ample evidence that inoculation with representatives of the *Bacillus* genus increases rice grain yield [68,69]. Similar results were obtained under greenhouse conditions upon inoculation with *Bacillus subtilis* C-3102, a producer of auxins with high protease activity, increasing grain yield (g pot^−1^) by 39.6% [70].

Regarding the harvest index, this parameter was higher in the co-inoculated treatments compared to the simple chemical inoculation treatments and those without inoculation. The harvest index is perhaps one of the parameters that best reflects the physiological balance in a crop and constitutes a variable factor in crop production, which is why a higher harvest index implies a physiological balance in the plant, producing more grain than chaff [71].

The co-inoculation with *B. siamensis* TUR07-02b and *P. megatherium* SMBH14-02 increased grain production. The synergy of both strains is due to their wide repertoire of PGP bacterial mechanisms through volatile compounds dependent on phytohormones [20]. On the other hand, *P. megaterium* (formerly *Bacillus megaterium*, [24]) has been widely described as an endophytic growth promoter of rice [15,21] and is involved in inducing systemic salicylic-acid-dependent resistance to fungal diseases in rice [15], protection against phytonematodes [72], degradation of insecticides [21], and even improvement of bacterial communities in the rhizosphere [15].

This is the first report wherein both *B. siamensis* and *P. megaterium* show synergy as growth promoters in rice under greenhouse conditions. The selected strains have activities that promote plant growth related to the production of auxins, solubilization of phosphates, nitrogen fixation, and production of cellulases and pectinases, which support the effectiveness of their inoculation on the improvement of the agronomic parameters evaluated. Furthermore, there is evidence that inoculation with selected strains of *Bacillus* and *Priestia* allows better efficiency in the use of mineral nitrogen present in the soil, mainly due to the positive regulation and increase in the expression of genes related to the transport of nitrates and amino acids in the rice plant [10,13]. It would explain the performance of the strains *B. siamensis* TUR07-02b and *P. megaterium* SMBH14-02 in promoting growth under different doses of nitrogen fertilization. This understanding will allow researchers in the future to select doses of fertilizers and apply individual or consortium inoculation with new strains, enabling new technologies to obtain integrated management of nutrients with a view to sustainable agriculture.

## 4. Materials and Methods

### 4.1. Strains under Study

The strains under study came from the strain collection of the Agricultural Microbiology Laboratory of the National University of San Martín. They corresponded to two species of *Bacillus* (two strains), one species of *Burkholderia* (two strains), and two species of *Priestia* (three strains), isolated from different organs of rice plants growing in fields of the Tumbes and San Martín regions in Peru [25] (Table 6).

The reference strains *Burkholderia vietnamiensis* la1a4 and la3c3 belonged to the Agricultural Microbiology collection of the National University of San Martín-Perú and were previously published as efficient strains in the production of PGPR mechanisms and growth promotion in rice [10]. The *Rhizobium tropici* strain CIAT 899 was donated by the strain collection of the Microbial Ecology Laboratory of the National Agrarian University La Molina-Peru.

### 4.2. Verification of Endophyte Colonization

The endophytic behavior of the strains under study was verified by recovering them from the interior of the tissue of 7-day-old seedlings [73]. Following previous confirmation of pure culture, each strain was cultivated overnight at 28 °C in 3 mL of Tryptone soybean broth (TSB) with shaking at 120 rpm. The inoculum was prepared from each cell pellet recovered by centrifugation (6000 rpm × 15 min) (Mikro200 Hettich, Germany) and then resuspended in a nitrogen-free Hoagland’s nutrient solution [74] containing the following (g L^−1^): 3.4 KH_2_PO_4_, 0.2 MgSO_4_. 7 H_2_O, 0.1 NaCl, 0.02 CaCl_2_, 0.0024 ZnSO_4_.7 H_2_O, 0.0024 Na_2_MoO_4_. 2H_2_O, 2.8 H_3_BO_3_, 0.0008 CuSO_4_.5 H_2_O, 0.0235 MnSO_4_.H_2_O, 4.5 KOH, 0.0049 FeCl_3_.6 H_2_O, and 0.015 mg EDTA, pH 7.0, until an OD_600nm_ of 1.0 (10^9^ CFU mL^−1^) was reached. Rice seeds were sterilized in 70% ethanol solution for 1 min after placing in 1% sodium hypochlorite for 5 min, followed by several washes with sterile water and germination in water agar (1%) at 25 °C for 24 h [75]. Germinated seeds without signs of contamination were sown in tubes containing a nutrient solution supplemented with agar (12 g L^−1^). Three days later, the seedlings were inoculated with 100 μL of each bacterial suspension. The experimental design consisted of nine treatments with five replicates: seven treatments corresponding to the strains under study, one positive control treatment inoculated with *Burkholderia vietnamiensis* la1a4, and a control treatment without inoculation. The treatments were subjected to photoperiod conditions of 14 h light 10 h^−1^ darkness at 28 °C ± 2 °C. After 7 days, seedlings were collected and separated into the root and aerial parts (stems and leaves). Each seedling was surface sterilized, and the disinfection efficiency was checked [73]. The plant tissue was crushed using saline solution (0.85%), and appropriate decimal dilutions of suspension in nutrient solution were surface sown on plates containing Tryptone soybean agar (TSA) and incubated at 30 °C for 48 h. The average CFU g^−1^ of root and fresh stem weight were obtained. Colonization tests were performed in triplicate.

### 4.3. Evaluation of Growth-Promoting Characteristics

#### 4.3.1. Determination of Indolic Compounds

The production of indolic compounds was determined by growing the strains overnight in 2 mL of TSB medium and incubating them at 30 °C [76]. The strains were inoculated in tubes containing 5 mL of TSB medium supplemented (150, 300, and 600 μg mL^−1^) or not supplemented with tryptophan (HI-media^®^, Maharashtra, India). The tubes were incubated at 30 °C at 180 rpm for 24 h. The fermented broth was centrifuged at 13,000 rpm for 3 min (Mikro200 Hettich, Tuttlingen, Germany). The supernatant was used to quantify the production of indolic compounds, mixing in triplicate in 0.5 mL of Salkowsky’s reagent [77] and 0.5 mL of supernatant, which were mixed by inversion and incubated in the dark for 20 min at room temperature. Following this, the absorbance at 535 nm was determined using a spectrophotometer (Thermofisher, Spectronic 200, Suwa, Japan), with a standard curve configured using indole acetic acid (50–1000 µg L^−1^) (Hi-media^®^, Maharashtra, India) and 0.5 mL of culture medium plus 0.5 mL of Salkowski’s reagent as a control. The results are expressed as μg IAA mL^−1^.

#### 4.3.2. Nitrogen Fixation

The determination of the nitrogen fixation capacity of endophytic bacteria was tested by culture in two semi-solid N-free medias, Burk medium (g L^−1^)—10.4 g Na_2_HPO_4_, 3.4 g KH_2_PO_4_, 26 mg CaCl_2_·2 H_2_O, 30 mg MgSO_4_, 0.3 mg MnSO_4_, 36 mg ferric citrate; 7.6 mg Na_2_MoO_4_·2 H_2_O, 10 µg p-aminobenzoic acid, 5 µg biotin, 4 g glucose, and 2 mM glutamate, pH 7.0 ± 0.2 [78], and JMV medium (g L^−1^)—5.0 g mannitol, 0.6 g K_2_HPO_4_, 1.8 g KH_2_PO_,_ 0.2 g MgSO_4_.7 H_2_O, 0.1 g NaCl, 0.02 CaCl_2_.2 H_2_O, 0.05 yeast extract, and 1.6 g agar-agar, pH 5.5–5.7 [79]. In both cases, forming a sub-film below the culture medium indicated nitrogen fixation.

#### 4.3.3. Solubilization of Ca, Fe, and Al Phosphates

The phosphate solubilization ability was evaluated by cultivating the strains in TSB medium and incubating at 30 °C for 24 h, adjusting the cell suspensions until an O.D._600nm_ of 1.0 was reached. Fifty-milliliters of glucose extract of yeast phosphate (GELP) broth, containing the required source of insoluble phosphate (Tricalcium phosphate), was inoculated with 1 mL of pre-inoculum, then incubated at 28 °C for 5 days with agitation at 130 rpm [80]. Finally, the supernatant was obtained by centrifugation at 13,500 rpm for 5 min (Mikro200 Hettich, Tuttlingen, Germany) and, with the help of a potassium phosphate standard curve (0.5–500 µg mL^−1^) (Merck ^®^, Darmstadt, Germany), the presence of quantified soluble phosphate was measured using the phosphomolybdate method [81], in which a blue complex develops, and its absorbance measured at 655 nm (Thermofisher, Spectronic 200, Suwa, Japan). Soluble phosphate values were expressed in µg mL^−1^ or mg L^−1^ [51]. The control strain *Rhizobium tropici* CIAT 899 was used to solubilize tricalcium phosphate, aluminum phosphate, and iron phosphate.

#### 4.3.4. Cellulase, Pectinase, and Chitinase Enzymes

For the measurement of hydrolytic enzymes, the strains were seeded in LB broth and incubated overnight at 37 °C with shaking at 150 rpm. Them, the cell pellet was washed and resuspended in sterilized LB medium to a final concentration of 1.0 (O.D._600nm_). Cellulase [82] and pectinase [83] activities were evaluated on carboxymethyl cellulose (CMC) agar and pectin agar, respectively. Five-microliters of each bacterial suspension was seeded in each culture medium and then incubated at 37 °C for 48 h. For the extracellular chitinase activity, 20 μL of each bacterial suspension was seeded on a plate containing colloidal chitin agar, which was incubated for 7 days at 30 °C. Enzymatic activities were evidenced according to the presence or absence of a solubilization halo.

### 4.4. Growth-Promotion Experiments

#### 4.4.1. Germination

Germination was evaluated by cultivating the strains in nutrient broth and incubating at 120 rpm for 48 h at 28 °C [84]. Cell suspensions were adjusted using a sterile saline solution (NaCl 0.89% *w*/*v*) at O.D._600nm_ = 1.0. The seeds of *O. sativa* var. “Bellavista” were sterilized in 70% ethanol for 1 min, 4% NaOCl for 6 min, and underwent six washes with sterile distilled water. For inoculation, the seeds were immersed in bacterial suspensions (seven strains) and sterile saline solution (0.89%) (control) for 1 h at room temperature. Each treatment consisted of 100 seeds inoculated in triplicate and planted in 1% water agar. Plates were sealed with parafilm and incubated in the dark for 72 h. The results were expressed in percentage of germination (%) = (number of germinated seeds/number of seeds sown) × 100 [85].

#### 4.4.2. Effects of Inoculation on Agronomic Parameters

For this test, the strains that showed the highest values of germination percentage concerning the control treatment, *B. siamensis* TUR07-02b, and *P. megaterium* SMBH14-02 were used. Under greenhouse conditions, a randomized complete block design (RCBD) was used, considering the following treatments: T1: *B. siamensis* TUR07-02b, T2: *P. megaterium* SMBH14-02, T3: co-inoculation of *B. siamensis* TUR07-02b and *P. megaterium* SMBH14-02, and T4: Nitrogenous chemical control treatment, by quintuplicate. Seeds were inoculated as the technique described above in a ratio (1:1) and sown at a rate of three seeds per pot (5 L). The soil was supplemented with 50, 75, and 100% of the required fertilizer nitrogen dose (180 kg N ha^−1^), and a 0% treatment corresponding to the basal nitrogen of the soil was considered. The plants were watered at will and maintained until grain formation under conditions of 27 ± 3 °C, with natural light. The following parameters were evaluated [10]: panicle length (mm), grains per panicle, panicles per plant, grain yield (g plant^−1^), straw yield (g plant^−1^), and harvest index (%).

### 4.5. Statistical Analysis

The data were evaluated according to their normality and homoscedasticity. Parametric and non-parametric ANOVA analyses were performed according to the results obtained in the different evaluations of growth promotion and biocontrol. Means were compared using the Tukey and Scott & Knott tests at a probability level of 5%. Data were reported as mean ± standard error, and the statistical program InfoStat was used.

## 5. Conclusions

The endophytic bacteria in this study expressed multiple growth-promoting characteristics at the “in vitro” level, being evaluated “in planta” at the gnotobiosis level under different levels of nitrogen fertilization. The selected *Bacillus* and *Priestia* strains are potential inoculants for rice plants, which must be tested and validated at the field level, evaluating different agronomic variables, such as number of applications, crop stage, and application dose, among others. In addition, sequencing the genome of both strains is necessary to elucidate their taxonomic affiliations and understand growth-promotion strategies, as well as other aspects that are still uncertain. Knowledge of these essential and applied aspects of *B. siamensis* TUR07-02b and *P. megaterium* SMBH14-02 as future commercial inoculants for rice will lead to the generation of new technologies in agriculture, reducing and optimizing the use of nitrogenous fertilizers in the cultivation of rice.

## Figures and Tables

**Table 1:** Log number of endophytic bacteria (CFU g^−1^) colonizing the root and stem tissue of *Oryza sativa* var. “Bellavista” growing for 7 days under gnotobiotic conditions.

Strains	Root	Stem
	Log 10 CFU g^−1^	Log 10 CFU g^−1^
*Priestia megaterium* SMBH14-02	3.52 (± 0.04) B	3.42 (± 0.01) C
*Priestia aryabhattai* SMNCH17-07	2.60 (± 0.02) C	3.04 (± 0.04) E
*Bacillus tequilensis* SMNCT17-02	5.71 (± 0.15) A	6.11 (± 0.03) A
*Priestia megaterium* TUT05-02	3.56 (± 0.13) B	3.25 (± 0.02) D
*Bulkholderia vietnamiensis* TUR04-03	3.86 (± 0.02) B	3.93 (± 0.01) B
*Bulkholderia vietnamiensis* TUR04-01a	3.76 (± 0.04) B	3.94(± 0.04) B
*Bacillus siamensis* TUR07-02b	2.99 (± 0.12) C	3.23 (± 0.01) D
*Bulkholderia vietnamiensis* la1a4 (Control)	3.58 (± 0.03) B	3.99 (± 0.01) B
Uninoculated Control	2.22 (± 0.08) C	2.99 (± 0.03) E
CV (%)	5.24	1.47

Mean and standard error (*n* = 3) followed by the same capital letter within the same column indicate differences between strains, with a probability of 5%, according to Tukey’s test (*p* < 0.05). CV: Coefficient of Variation.

**Table 2:** Evaluation of the production of indole compounds by endophytic rice bacteria at four concentrations of supplemented tryptophan (μg IAA mL^−1^).

Strains	ppm (mg L^−1^)			
	0	150	300	600
*P. megaterium* TUT05-02	45.43 (± 1.52) B^c^	77.69 (± 11.75) A^b^	144.78 (± 1.45) A^a^	85.75 (± 3.93) C^b^
*Bacillus siamensis* TUR07-02b	40.27 (± 6.27) B^c^	62.31 (± 11.58) AB^bc^	123.17 (± 5.59) Ba	98.44 (± 8.77) B^ab^
*B. vietnamiensis* TUR04-01a	26.94 (± 1.04) CD^b^	12.10 (± 3.00) D^b^	50.48 (± 7.39) E^a^	28.44 (± 2.79) F^b^
*p. megaterium* SMBH14-02	58.33 (± 4.30) A^b^	51.45 (± 5.14) B^b^	71.56 (± 2.73) D^b^	335.65 (± 6.45) A^a^
*B. vietnamiensis* TUR04-03	36.08 (± 2.08) BC^b^	40.38 (± 4.72) BC^b^	75.97 (± 6.06) D^a^	43.28 (± 3.36) E^b^
*Bacillus tequilensis* SMNCT17-02	19.73 (± 1.37) CD^b^	25.00 (± 5.29) CDE^b^	111.67 (± 4.57) B^a^	2.20 (± 0.47) G^c^
*P. aryabhattai* SMNCH17-07	18.01 (± 0.88) D^b^	ND	57.69 (± 2.17) DE^a^	10.27 (± 1.51) G^c^
*B. vietnamiensis* la3c3 (Control)	18.55 (± 3.57) D^b^	21.02 (± 5.27) CD^b^	ND	64.57 (± 5.28) D^a^
CV (%)	16.19	27.17	10.29	9.27

Mean and standard error (*n* = 3) followed by the same letter are not significant at 5% probability by the Tukey test (*p* < 0.05). Capital letters indicate differences between strains, and superscript lowercase letters indicate differences between levels of tryptophan supplementation. ND: Not determined, CV: Coefficient of Variation.

**Table 3:** Evaluation of plant-growth-promoting bacterial characteristics.

Strains	Phosphate Solubilization (µg mL^−1^)			Fixation of Nitrogen		Production of Extracellular Enzymes		
	Ca-P	Fe-P	Al-P	Burk	JMV	Cellulases	Pectinases	Chitinases
*P. megaterium* TUT05-02	0	0	0	+	+	-	-	-
*Bacillus siamensis* TUR07-02b	0	0	0	+	+	-	+	-
*B. vietnamiensis* TUR04-01a	0	0	0	+	+	-	-	-
*P. megaterium* SMBh14-02	20.94 A	0	0	+	+	+	+	-
*B. vietnamiensis* TUR04-03	0	0	0	+	+	-	-	-
*Bacillus tequilensis* SMNCT17-02	0	0	7.15 A	+	+	-	-	-
*P. aryabhattai* SMNCH17-07	6.84 B	0	0	+	+	+	+	-
*R. tropici* CIAT 899 (Control)	2.56 B	4.56 A	3.42 B	ND	ND	ND	ND	ND
*B. vietnamiensis* la3c3 (Control)	ND	ND	ND	+	+	ND	ND	ND
CV (%)	10.41	3.49	4.21	-	-	-	-	-

Mean and standard error (*n* = 3) followed by the same capital letter within the same column indicate differences between strains with respect to phosphate solubilization, with a probability of 5%, according to Tukey’s test (*p* < 0.05). +: Halo presence, -: No halo, ND: Not determined, CV: Coefficient of Variation, Burk and JMV: Semi-solid N-free media.

**Table 4:** Effect of rice endophytic bacteria on the germination of *Oryza sativa* var. “Bellavista”.

Strains	Germination (%)
*P. megaterium* SMBH14-02	93.00 (± 1.00) A
*Bacillus siamensis* TUR07-02b	92.00 (± 1.63) A
*B. vietnamiensis* TUR04-01a	91.00 (± 1.00) A
*B. vietnamiensis* TUR04-03	91.00 (± 1.00) A
*P. megaterium* TUT05-02	90.00 (± 2.00) A
*Bacillus tequilensis* SMNCT17-02	85.37 (± 2.13) B
*P. aryabhattai* SMNCH17-07	84.00 (± 3.27) B
Uninoculated Control	82.00 (± 5.29) B
CV (%)	5.77

Mean and standard error (*n* = 3) followed by the same capital letter indicate differences between strains with respect to germination, with a probability of 5%, according to Tukey’s test (*p* < 0.05). CV: Coefficient of Variation.

**Table 5:** Effect of the inoculation of endophytic *B. siamensis* and *P. megaterium* on the agronomic parameters of *Oryza sativa* var. “Bellavista” growing under greenhouse conditions.

Nitrogen Dose (%) *	*B. siamensis* TUR07-02b	*P. megaterium* SMBH14-02	*B. siamensis* TUR07-02b + *P. megaterium* SMBH14-02	Chemical Control	CV (%)
Panicle length (mm)					
0	29.43 (± 0.21)A^a^	29.58 (± 0.46) A^a^	28.68 (± 0.26) B^a^	24.87 (± 1.06) A^b^	6.78
50	29.42 (± 1.03) A^a^	28.36 (± 0.60) A^a^	30.38 (± 0.52) A^a^	25.01 (± 0.61) A^b^	8.01
75	28.56 (± 0.33) A^b^	28.28 (± 0.57) A^b^	30.90 (± 0.67) A^a^	24.72 (± 0.32) A^c^	5.57
100	26.82 (± 1.10) A^b^	28.69 (± 0.43) A^b^	32.27 (± 0.59) A^a^	24.41 (± 0.76) A^c^	8.60
CV (%)	8.64	5.70	5.52	9.43	7.34
Grains per panicle					
0	89.30 (± 3.12) A^a^	87.50 (± 3.86) A^a^	83.20 (± 3.34) C^a^	80.20 (± 5.58) B^a^	13.82
50	83.40 (± 2.45) A^b^	85.70 (± 3.88) A^a^	115.70 (± 6.43) B^a^	88.60 (± 3.33) B^b^	14.28
75	87.80 (± 2.53) A^c^	77.90 (± 3.04) B^c^	150.80 (± 4.78) A^a^	115.40 (± 4.93) A^b^	16.48
100	70.20 (± 5.38) B^b^	73.80 (± 4.20) B^b^	129.20 (± 4.40) B^a^	67.50 (± 9.47) B^b^	16.06
CV (%)	13.67	14.67	12.85	20.12	15.41
Panicles per plant					
0	2.40 (± 0.24) A^a^	2.20 (± 0.20) A^a^	2.20 (± 0.20) A^a^	2.37 (± 0.00) A^a^	19.02
50	2.20 (± 0.20) A^a^	2.40 (± 0.24) A^a^	2.60 (± 0.24) A^a^	2.00 (± 0.00) A^a^	19.44
75	2.20 (± 0.20) A^a^	2.00 (± 0.00) A^a^	2.20 (± 0.20) A^a^	3.17 (± 0.24) A^a^	19.02
100	1.80 (± 0.20) A^a^	2.20 (± 0.20) A^a^	1.60 (± 0.24) A^a^	3.63 (± 0.24) A^a^	17.78
CV (%)	22.06	19.02	23.26	19.36	21.05
Grain yield (g plant^−1^)					
0	3.69 (± 0.36) A^a^	3.16 (± 0.36) A^a^	2.78 (± 0.28) B^a^	2.71 (± 0.33) A^a^	24.15
50	2.67 (± 0.42) A^a^	3.50 (± 0.39) A^a^	4.06 (± 0.12) A^a^	3.09 (± 0.26) A^a^	21.55
75	2.70 (± 0.36) A^b^	2.64 (± 0.18) A^b^	4.34 (± 0.16) A^a^	3.11 (± 0.36) A^b^	19.67
100	1.90 (± 0.21) A^b^	2.72 (± 0.32) A^a^	3.42 (± 0.34) B^a^	1.56 (± 0.17) B^b^	25.06
CV (%)	28.35	24.00	14.72	24.64	22.51
Straw yield (g plant^−1^)					
0	16.45 (± 0.59) A^b^	21.72 (± 3.06) A^a^	13.91 (± 0.89) A^b^	10.87 (± 0.80) A^b^	23.72
50	16.66 (± 1.14) A^a^	17.84 (± 0.80) A^a^	15.10 (± 1.44) A^a^	14.02 (± 1.39) A^a^	17.14
75	18.15 (± 1.79) A^a^	15.25 (± 1.15) A^a^	15.28 (± 0.57) A^a^	12.85 (± 0.70) A^a^	18.57
100	10.77 (± 1.60) B^a^	16.17 (± 2.51) A^a^	12.67 (± 0.79) A^a^	10.74 (± 1.32) A^a^	19.80
CV (%)	19.64	26.48	15.33	20.21	21.77
Harvest index (%)					
0	18.23 (± 1.37) A^a^	13.08 (± 1.08) A^a^	16.69 (± 1.40) B^a^	20.01 (± 0.50) A^a^	21.06
50	13.75 (± 1.71) A^a^	16.46 (± 1.90) A^a^	21.66 (± 1.61) A^a^	18.48 (± 0.38) A^a^	23.10
75	12.89 (± 1.01) A^b^	14.95 (± 1.23) A^b^	22.16 (± 0.56) A^a^	19.70 (± 0.06) A^a^	19.53
100	16.04 (± 2.72) A^a^	15.07 (± 2.05) A^a^	21.20 (± 1.53) A^a^	13.34 (± 0.90) A^a^	18.93
CV (%)	26.65	24.36	14.69	17.86	23.28

Mean and standard error (*n* = 3) followed by the same letter are not significant at 5% probability by the Scott & Knott test (*p* < 0.05). Capital letters indicate the differences at the nitrogen dose level in each agronomic parameter, and lowercase letters in superscript indicate the statistical differences at the strain level in each nitrogen dose. *: Complete dose 180 kg N ha^−1^, CV: Coefficient of Variation.

**Table 6:** Origin and taxonomic affiliation using the 16S rRNA gene of endophytic rice strains, adapted from [25].

Species	Place of Origin	Isolation Organ	Similar Type Strain	Similarity Percentage (%)	Number of Nucleotides
*P. aryabhattai* SMNCH17-07	Nueva Cajamarca, San Martín	leaf	B8W22^T^	100.00	1416
*P. megaterium* SMBH14-02	Bellavista, San Martín	Leaf	NBRC15308^T^	99.79	1438
*B. tequilensis* SMNCT17-02	Nueva Cajamarca, San Martín	Stem	10b^T^	99.65	1437
*P. megaterium* TUT05-02	Tumbes	Stem	NBRC15308^T^	100.00	1403
*B. vietnamiensis* TUR04-03	Tumbes	Root	LMG10929^T^	99.93	1403
*B. vietnamiensis* TUR04-01a	Tumbes	Root	LMG10929^T^	99.93	1416
*B. siamensis* TUR07-02b	Tumbes	Root	KCTC13613^T^	99.78	1389

## Data Availability

Data used for analysis can be obtained through the corresponding author, Rios-Ruiz W.F. (wrios@unsm.edu.pe).
